# Unravelling the thread of *Podarcis* omics; insights into the genome and transcriptome of the Cretan wall lizard

**DOI:** 10.1007/s13258-025-01676-1

**Published:** 2025-10-25

**Authors:** Manos Stratakis, Panagiotis Ioannidis, Iliana Bista, Dominic Absolon, Will Eagles, Shane McCarthy, Amy Denton, Petros Lymberakis, Nikos Poulakakis

**Affiliations:** 1 Department of Biology, School of Sciences and Engineering, University of Crete, Heraklion, Greece; 2 Natural History Museum of Crete, School of Sciences and Engineering, University of Crete, Heraklion, Greece; 3 Institute of Computer Science (ICS), Foundation for Research and Technology – Hellas (FORTH), Heraklion, Greece; 4 Senckenberg Research Institute and Natural History Museum, 60325 Frankfurt am Main, Germany; 5 Wellcome Sanger Institute, Tree of Life, Wellcome Genome Campus, Hinxton, CB10 1SA UK

**Keywords:** *Podarcis cretensis*, Reference genome, Comparative genomics, Transcriptomics, Gene set, Squamata

## Abstract

**Background:**

Whole genome data are invaluable resources for both conservation and adaptation studies, especially for endemic species, providing insights into the evolution of genes involved in genomic adaptation across different environments.

**Objective:**

We compare the newly generated genomic and transcriptomic data of the Cretan endemic lizard species *Podarcis cretensis* to other Podarcis species to obtain an overview of gene family evolution and genome structure within the genus.

**Methods:**

Comparative genomic and transcriptomic analyses were performed using the newly published genome of *P. cretensis*. A gene set was predicted using RNA-seq data from 36 samples, comprising three tissues (liver, brain, and muscle) from both male and female individuals across three distinct habitats.

**Results:**

The main findings revealed that *P. cretensis* and *P. raffonei* present the best genome assemblies and the most syntenic among the *Podarcis* species examined. Moreover, *P. cretensis* displayed the highest percentage of single-copy genes and the lowest percentage of duplicated genes. These duplicated genes are primarily associated with immune and sensory-related gene families, including chemokines, interleukins, immunoglobulin-like domain proteins, secreted proteins, and vomeronasal type-2 receptors.

**Conclusions:**

This study deepens our understanding of chromosome structure, gene expression, and genome evolution in the *Podarcis* genus, representing the most extensive comparative analysis to date. The newly predicted gene set of the insular endemic species *P. cretensis* offers initial insights into gene expression related to adaptation across environments and tissues. Comparative genomic analyses further revealed gene families potentially involved in environmental adaptation.

**Supplementary Information:**

The online version contains supplementary material available at 10.1007/s13258-025-01676-1.

## Introduction

Lizards of the *Podarcis* genus can serve as useful model species for evolutionary studies because of their reduced dispersal capability and ability to adapt to diverse environments (Spilani, Bougiouri et al. 2019, Pafilis, Adamopoulou et al. 2024). By examining their genomes, we gain valuable information about the interplay among palaeogeography, the environment, and natural selection, which has played a crucial role in shaping the observed genetic diversity within this group (Spilani, Bougiouri et al. 2019). In addition, their ability to adapt to different altitudinal and thermal environments is invaluable for studying the effects of climate change on species adaptation (Taylor et al. 2021).

The continuous decrease in sequencing cost as well as the development of faster and more efficient bioinformatics tools over recent decades has facilitated the exploration of the genetic basis of phenotypic plasticity, diversity and adaptation. Genome-wide and whole transcriptome analysis, contributes to understanding of how organisms can rapidly respond to environmental changes. In this context, reference genomes have played a crucial role in conservation genomics, enabling the assessment of genetic diversity, gene expression-based exploration of adaptation mechanisms, and characterization of deleterious mutations (Formenti, Theissinger et al. 2022). A reference genome also acts as a comprehensive record of an organism's coding sequences and other functional DNA elements, serving as a foundation for various biological studies (Whibley, Kelley et al. 2021), and, from a wider perspective, facilitates monitoring and managing diversity across whole ecosystems (Theissinger, Fernandes et al. 2023). Even if there has been a significant acceleration in the production of highly contiguous genomes in recent years, certain clades, such as squamate reptiles, are still underrepresented (Pinto, Keating et al. 2022, Card, Jennings et al. 2023). Moreover, genes related to environmental adaptation are generally understudied in ectothermic vertebrates (Diele-Viegas and Rocha 2018, Wollenberg Valero, Garcia‐Porta et al. 2022).

Recently, studies on the genomes and transcriptomes of other *Podarcis* species, including *P. muralis* (Andrade, Pinho et al. 2019), *P. siculus* (Trapanese, Buglione et al. 2017), *P. raffonei* (Gabrielli, Benazzo et al. 2023) and *P. lilfordi* (Gomez-Garrido, Cruz et al. 2023), have been published. These studies showed that a high-quality genome, especially for an endemic species, is crucial not only for basic research but also for applied conservational studies, such as in the case of *P. raffonei* (Gabrielli, Benazzo et al. 2023; Salvi 2023). A reference genome can also provide useful insights in understanding different aspects of chromosomal evolution. For example in recent years, there has been growing discussion around microchromosomes, as -despite the lack of a clear definition- they exhibit distinct characteristics compared to macrochromosomes (Perry, Schield et al. 2021, Srikulnath, Ahmad et al. 2021) and have never explicitly examined in taxa like geckos and lacertids (Pinto et al. 2023).

The endemic Cretan wall lizard, *Podarcis cretensis* (Wettstein, 1952) (Squamata: Lacertidae), is an ideal organism for both ecophysiological and evolutionary adaptation studies related to climate change. The Cretan wall lizard, one of the two endemic reptile species of Crete and the surrounding islets (Lymberakis, Pafilis et al. 2018), was previously considered a subspecies of *P. erhadii*, but it was eventually recognized as a separate species (Lymberakis, Poulakakis et al. 2008). The species presents an unusual residual distribution, as it is dispersed throughout western Crete, whereas to the east, it is found only on islets surrounding the main island. Populations of the species can also be found from coastal areas and islets with few hectares of surface to altitudes exceeding 2100 m. To date, several studies have been carried out on this species, including studies of its phylogeny (Poulakakis, Lymberakis et al. 2003, Poulakakis, Lymberakis et al. 2005), population structure (Spilani, Bougiouri et al. 2019, Psonis, Antoniou et al. 2021), distribution (Baral 2019) and body development (Tomczak, Sontheimer et al. 2012). Recently, the thermal biology and genetic adaptation of different populations of this species were investigated from our group via genomic and transcriptomic data as part of ongoing work, highlighting the need for a reference genome, as reference-based methods outperform de novo methods in both genomic and transcriptomic profiling (Guo, Ma et al. 2021, Lee, Na et al. 2021). Moreover, a new reference genome can be used in comparative analyses, providing insights into chromosome evolution, particularly in key topics of squamate reptilian genomics, such as microchromosomes structure and sex determination system evolution (Pinto et al. 2023).

Here, we present an in-depth analysis of the published reference genome assembly of *P. cretensis* (Poulakakis, Lymberakis et al. 2024). Different approaches of comparative genomics analyses were undertaken against 10 other available *Podarcis* genome assemblies, together with a synteny analysis. A new gene set was predicted for *P. cretensis* using RNA-seq data derived from 36 samples, comprising three different tissues (brain, liver, muscle), both sexes and individuals from three distinct habitats to capture variability across environmental conditions. The gene set was then compared against published annotations from four other *Podarcis* species. Orthology analysis was used to identify orthologues and single-copy genes in *P. cretensis* and a gene expression atlas was produced, which was deposited in a public repository to aid the respective research community. Finally, an initial analysis of the expression levels of populations adapted in different environmental conditions is presented.

## Material & methods

### Genomic DNA extraction and sequencing

An adult male *P. cretensis* collected from the Rousies highlands over Anopoli village in the Lefka Ori mountains (Supplementary Figure SA1) was used for the rPodCre2.1 genome assembly (Poulakakis, Lymberakis et al. 2024). The specimen was moved to the Natural History Museum of Crete (NHMC) facilities and euthanized by concussion in compliance with the European Commission directive (Close, Banister et al. 1996). Tissue samples from the brain, liver, and heart were immediately stored at -80 °C, while the remaining parts were preserved in 96% ethanol (collection number NHMC80.3.51.2950).

The tissue samples were sent at the Wellcome Sanger Institute (WSI) Tree of Life Core Laboratory, where DNA extraction and sequencing were performed. In addition, RNA from muscle was extracted for RNAseq. Briefly, HMW DNA was extracted via the automated MagAttract v2 protocol (Oatley, Denton et al. 2023), followed by shearing and purification. The concentration was assessed via Nanodrop and Qubit dsDNA high-sensitivity assay kits, and the fragment profile was evaluated via the FemtoPulse system. For more details, see Poulakakis, Lymberakis et al. (2024).

### Genome assembly and curation

The genome assembly was created using 27 × coverage of PacBio sequencing data and Arima2 Hi-C data and HiGlass (Kerpedjiev, Abdennur et al. 2018). Briefly, primary assembly was performed with Hifiasm via PacBio data (Cheng, Grueber et al. 2022) and scaffolded with Hi-C data (Rao, Huntley et al. 2014) via YaHS (Zhou, McCarthy et al. 2023). Decontamination and correction were performed according to (Howe et al. 2021). Manual curation was performed via HiGlass (Kerpedjiev, Abdennur et al. 2018) and PretextView (Harry 2022). k-mer completeness and QV were calculated in Merqury (Rhie, Walenz et al. 2020). The genome was analysed within the BlobToolKit environment (Challis, Richards et al. 2020), and BUSCO scores (Simão, Waterhouse et al. 2015, Manni, Berkeley et al. 2021) were calculated. For more details, see Poulakakis, Lymberakis et al. (2024). The above work was conducted by the Tree of Life Programme at the Wellcome Sanger Institute (https://www.sanger.ac.uk/programme/tree-of-life/) as a component of the pilot project for the European Reference Genome Atlas (Mc Cartney, Formenti et al. 2024).

### Population selection and temperature treatments

The samples were selected to represent all the different *P. cretensis* ecotypes. Specifically, four individuals were taken from the islet of Mikronisi Chrysis, four from the Therisos gorge located at an altitude of 1000 m, and four from the Rousies Plateau in the White Mountains, located at an altitude greater than 2000 m (Supplementary Figure SA1). Additionally, we conducted a crossover adaptation experiment with two individuals from Mikronisi Chrysis and two from the Rousies Plateau. These individuals were acclimated for one month prior to RNA extraction, with those from Mikronisi placed in a 4 °C environment temperature cold room to simulate winter temperature conditions typical of Rousies and those from Rousies housed in an open-air room (RT) simulating Mikronisi winter temperatures (Supplementary Table SA2).

### RNA extraction, library preparation and sequencing

Total RNA was extracted from three tissues (brain, liver and muscle) from 12 individuals (Supplementary Table SA2), which were preserved in RNAlater™ (Invitrogen™) and stored at -20 °C. The brain and liver are among the highest energy-expending organs in the body, and their metabolism is known to vary depending on temperature conditions, suggesting that these organs are important thermoregulatory organs (Akashi, Cádiz Díaz et al. 2016). Muscles, on the other hand, are also important for studying adaptive mechanisms under different conditions, as the locomotion of lizards varies greatly under different thermal conditions and facilitates most behaviours (Smith, Anderson et al. 2022). The monophasic reagent TRItidy G™ (ITW Reagents) was used for the extraction, according to the recommended protocol from the manufacturer. The RNA was quantified with a Qubit fluorometer, and 1 μg of RNA from each sample was used for the construction of cDNA libraries following the TruSeq Stranded mRNA protocol (Illumina). Sequencing was performed on Illumina HiSeq 2000 (Illumina Inc., San Diego, California, USA) with a 150 bp paired-end protocol.

### Transposon annotation and repeat masking

De novo identification of repeat elements was carried out via RepeatModeller v2.0.4 (Smit and Hubley 2023a), with the parameter “-LTRStruct”. The list of identified repeats was searched against SwissProt (Boutet, Lieberherr et al. 2016) via DIAMOND v2.1.4.158 (Buchfink, Reuter et al. 2021), and sequences that were similar to proteins not related to repeat elements were removed. Additional repeats were found via the de novo assembly of the RNA-seq reads from the brain, liver and muscles that were generated in the frame of this study. De novo transcriptome assembly was generated with Trinity v2.12.0 (Grabherr, Haas et al. 2011) using the default parameters. TransDecoder v5.7.0 (Haas, BJ. https://github.com/TransDecoder/TransDecoder v5.7.0) was then used with default parameters to predict genes in the assembled transcripts, and hmmscan from the HMMer 3.1b2 package (HMMER.org) was used to predict Pfam domains (Mistry, Chuguransky et al. 2021) in the amino acid sequence of the predicted genes. Transcripts that contained domains related to transposable elements were extracted from the hmmscan output files via a custom Python script. The repeat models identified with RepeatModeler and those identified from the RNAseq data were combined for masking. Repeat masking in the genome assembly was performed via RepeatMasker v4.1.4 (Smit and Hubley 2023b) with the parameters “-xsmall”. The repeat library consisted of the abovementioned RepeatModeler/RNAseq species-specific repeats and the conserved repeats in the Dfam v3.7 database (Storer, Hubley et al. 2021).

### Genome assemblies and gene annotations used

The *P. cretensis* genome assembly (rPodCre2.1) was obtained from GenBank via NCBI dataset tools (NCBI 2024). The quality of the resulting assembly and 10 additional genomes and annotations of other *Podarcis* species (*P. lilfordi*, *P. muralis*, *P. raffonei*) was assessed with BUSCO v5.4.7 (Waterhouse, Seppey et al. 2018) using the Sauropsida set (Table 1). The CDS per gene ratio was calculated from the available gff files of published annotations rPodLil1.2, PodMur_1.0 and rPodRaf1.pri respectively.

**Table 1:** Comparative genome statistics for 11 *Podarcis* species genomes

Species	Accession	Genome size (Mbp)	Contig N50 (Mbp)	Scaffold N50 (Mbp)	Scaffold number	BUSCO^1^ (genome)	BUSCO^1^ (gene annotation)
*P. cretensis*	GCA_951804945.1	1.508	65.7	94.6	50	C:97.8%	C:94.8
						[S:96.3%,D:1.5%]	[S:92.7%,D:2.1%]
						F:0.3%,M:2.0%	F:0.6%,M:4.7%
						E:2.5%	
*P. muralis*	GCF_004329235.1	1.51	0.7146	92.4	2,16	C:96.5%	C:96.8%
						[S:93.6%,D:2.9%]	[S:43.6%,D:53.2%]
						F:0.6%,M:2.9%	F:0.7%,M:2.6%
						E:2.1%	
*P. raffonei*	GCF_027172205.1	1.51	64.1	93.6	27	C:97.8%	C:98.4%
						[S:96.0%,D:1.8%]	[S:43.2%,D:55.3%]
						F:0.3%,M:1.9%	F:0.2%,M:1.3%
						E:2.4%	
*P. lilfordi*	GCA_947686815.1	1.46	1.5	90	2,148	C:97.7%	C:94.7%
						[S:96.0%,D:1.6%]	[S:61.4%,D:33.3%]
						F:0.3%,M:2.0%	F:1.2%,M:4.1%
						E:2.4%	
*P. siculus*	GCA_964188175.1	1.571	3.1	94.5	293	C:97.7%	N/A
						[S:96.0%,D:1.7%]	
						F:0.3%,M:1.9%	
						E:2.4%	
*P. erhardii*	GCA_964252035.1	1.496	3.2	93.5	325	C:95.2%	N/A
						[S:93.7%,D:1.5%]	
						F:0.3%,M:4.5%	
						E:2.4%	
*P. melisellensis*	GCA_964234825.1	1.437	3.1	92.1	200	C:94.6%	N/A
						[S:93.2%,D:1.4%]	
						F:0.4%,M:5.0%	
						E:2.4%	
*P. filfolensis*	GCA_964270895.1	1.507	3	91.6	348	C:97.7	N/A
						[S:95.9%,D:1.8%]	
						F:0.3%,M:2.0%	
						E:2.4%	
*P. gaigeae*	GCA_964106915.2	1.515	2.9	91.9	210	C:97.7%[S:96.1%,D:1.6%]	N/A
						F:0.3%,M:2.0%	
						E:2.4%	
*P. bocagei*	GCA_964188305.1	1.615	2.7	94.9	506	C:96.3	N/A
						[S:94.4%,D:1.9%]	
						F:0.4%,M:3.3%	
						E:2.3%	
*P. pityusensis*	GCA_964106635.2	1.417	1.7	92.4	183	C:94.5	N/A
						[S:93.0%,D:1.5%]	
						F:0.5%,M:5.1%	
						E:2.3%	

^1^BUSCO score is based on the Sauropsida_odb10 BUS CO set, which contains n = 7,480 BUSCOs. C = complete [S = single copy, D = duplicated], F = fragmented, M = missing, E = containing internal stop codons.

### Gene annotation in P. cretensis

To predict genes in the *P. cretensis* genome assembly, the BRAKER pipeline v3.0.2 (Brůna, Hoff et al. 2021) was used in ETP mode with default parameters. RNAseq Illumina libraries from the brain, liver and muscle tissues of different *P. cretensis* individuals and populations were used as transcription evidence (Supplementary Table 2). Reads were first mapped to the *P. cretensis* genome with Hisat v2.2.1 (Kim, Paggi et al. 2019) with the parameters “—dta-cufflinks”. The generated SAM files were converted to sorted BAM files with SAMtools v1.16.1 (Danecek, Bonfield et al. 2021) with default parameters. Additionally, the vertebrate proteomes from OrthoDB v10 (Kriventseva, Kuznetsov et al. 2019) were used as one source of protein evidence. An additional source of protein evidence was obtained from the peptides predicted in the Trinity assemblies with TransDecoder (mentioned in the previous section). These two sources of protein evidence were pooled together, and sequences that were > 97% similar to each other were removed using CD-HIT v4.7 (Fu, Niu et al. 2012) to reduce redundancy. The final file with all protein evidence was passed as protein evidence to BRAKER with the “–prot_seq” parameter. Several gene sets were produced with BRAKER via a different combination of RNAseq and protein evidence, and each set was evaluated with BUSCO v5.4.7 and the sauropsidan set. Ideally, the best gene set is the one for which the number of complete BUSCOs is greater than that of the genome assembly. The functional annotation of the predicted genes was performed by searching for significant similarities in the amino acid sequences of the genes against the Uniref50 protein database. DIAMOND v2.1.4.158 (Buchfink, Reuter et al. 2021) was used in these similarity searches with the default parameters. Custom Python scripts were used to extract information on the gene density and gene content of each chromosome. As gene prediction is a time-consuming and resource-intensive process we deposited the herein generated gene set in a public repository in order to aid researchers in the relevant field.

### Expression atlas

The gene expression levels of the predicted genes were estimated via Salmon v.1.10.3 (Patro, Duggal et al. 2017) with the *P. cretensis* genome sequence (rPodCre2.1) as the decoy sequence in mapping-based mode as described in Salmon v.1.10.3 documentation. Gene expression levels were calculated using all the available RNAseq reads from the brain, liver and muscle, providing an initial characterization of the expression profile of *P. cretensis*.

### Orthology analysis

Orthology analysis was conducted via OrthoFinder (Emms and Kelly 2019) to identify homologous relationships between four *Podarcis* species: *P. cretensis* (present study), *P. raffonei* (Gabrielli, Benazzo et al. 2023), *P. muralis* (Andrade, Pinho et al. 2019), *P. lilfordi* (Gomez-Garrido, Cruz et al. 2023), and *Anolis carolinensis* (Alföldi, Di Palma et al. 2011). A given rooted species tree according to the whole-genome phylogenetic tree of *Podarcis* (Yang, Feiner et al. 2021) was used for orthology analysis, with the species *Anolis carolinensis* used as an outgroup.

#### Synteny analysis and microchromosome detection

Pairwise synteny analysis between *P. cretensis* and eight *Podarcis* assemblies was conducted after pairwise genome alignment via Minimap2 (Li 2018). Synteny analysis and dot plot visualization were generated with the D-GENIES online tool (Cabanettes and Klopp 2018) with the 'Repeatedness' parameter set to 'Many repeats’. The results were further analysed and visualized with custom Python scripts. For microchromosome detection, we examined the GC content, total length and gene density of the chromosome-like scaffolds. The exact number of chromosome rearrangements between *P. cretensis* and the other *Podarcis* was extracted from resulting PAF files.

## Results

### Comparison with the genomes of other Podarcis lizards

The *P. cretensis* genome assembly (rPodCre2.1) has a total size of 1.5 Gb, with a scaffold N50 of 94.6 Mb, QV 65.1% and k-mer completeness 100.0%. The genome size is similar to that of other published *Podarcis* genomes (Table 1). The BUSCO completeness score for the genome assemblies ranged from 94.5% to 97.8%. *P. cretensis* and *P. raffonei* presented the highest completeness (Table 1). Both synteny analysis and Hi-C data (Poulakakis, Lymberakis et al. 2024) revealed 19 chromosome-like scaffolds (18 autosomes and a Z sex chromosome in *P. cretensis*), which is consistent with the typical karyotype of *Podarcis* (Capula, Nascetti et al. 1982, Naveira, Rojo et al. 2023).

**Fig. 1:**
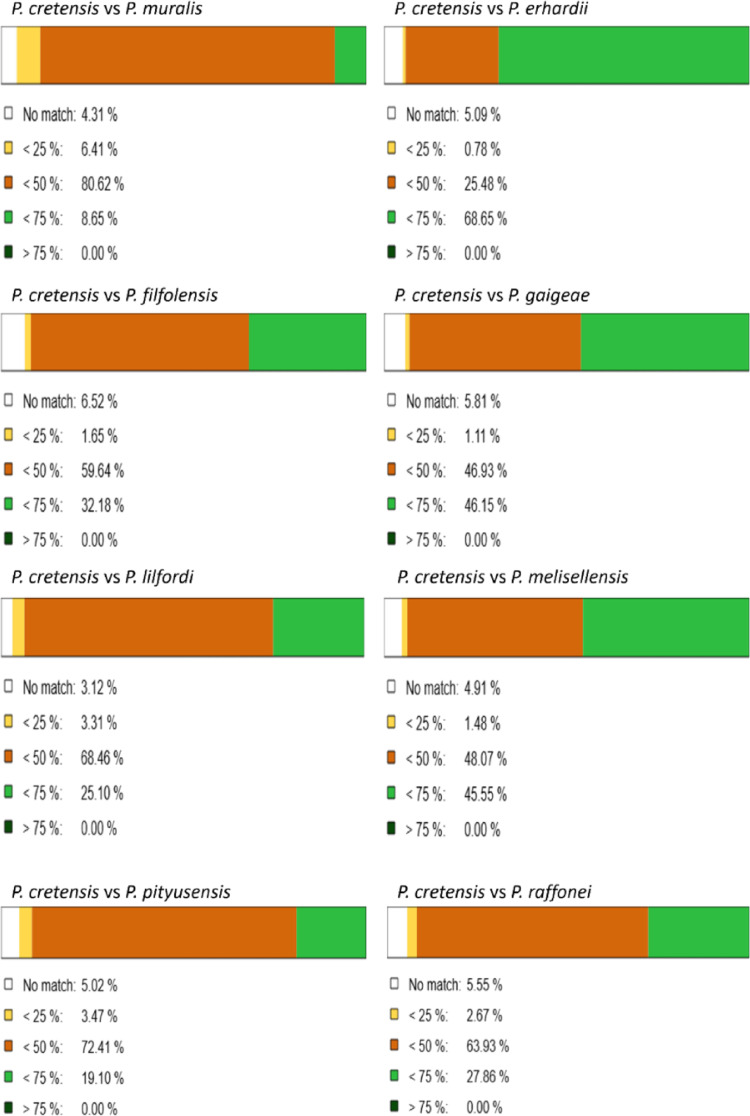
Similarity levels of pairwise comparisons between P. cretensis and the other Podarcis genome alignments as resulted from D-GENIES

**Fig. 2:**
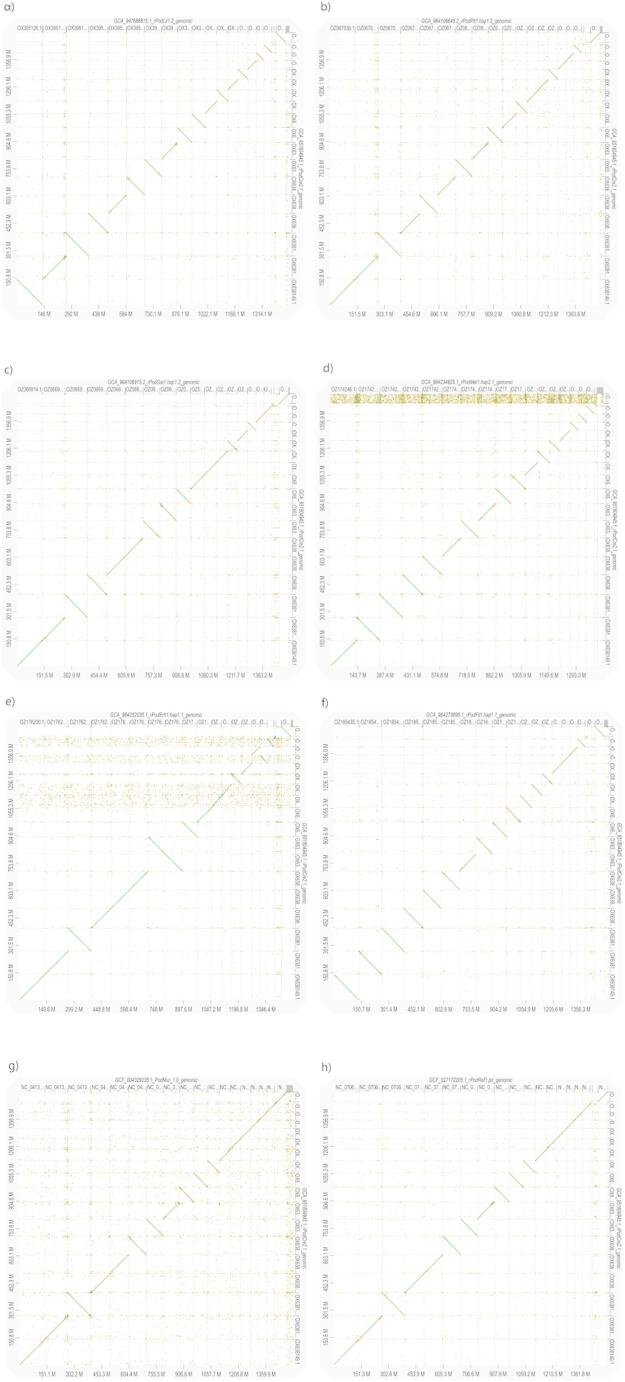
Dot plots resulting from pairwise synteny analysis generated by D-genies from eight *Podarcis* species against *P. cretensis*. **a ***P. filfolensis* (rPodFil1.hap1.1)—*P. cretensis* (rPodCre2.1), **b ***P. erhardii* (rPodErh1.hap1.1)—*P. cretensis* (rPodCre2.1), **c** P. raffonei (rPodRaf1—*P. cretensis* (rPodCre2.1), **d** P. muralis (PodMur_1.0)—*P. cretensis* (rPodCre2.1), **e **P. lilfordi (rPodLil1.2)—*P. cretensis* (rPodCre2.1), **f **P. pityusensis (rPodPit1.hap1.2)—*P. cretensis* (rPodCre2.1), **g ***P*. *gaigeae* (rPodGai1.hap1.2)—*P. cretensis* (rPodCre2.1), **h** P. melisellensis—*P. cretensis* (rPodCre2.1). The diagonal lines in each plot indicate regions of high synteny, the colors correspond to similarity values [deep green high similarity (> 75%), green medium high similarity (50–75%), orange medium low similarity (25–50%), yellow low similarity (0–25%)], the flipped lines indicate chromosomal inversion, the discontinuities indicate insertions or deletions, translocations and rearrangements, and the scattered dots indicate sequence divergence

**Fig. 3:**
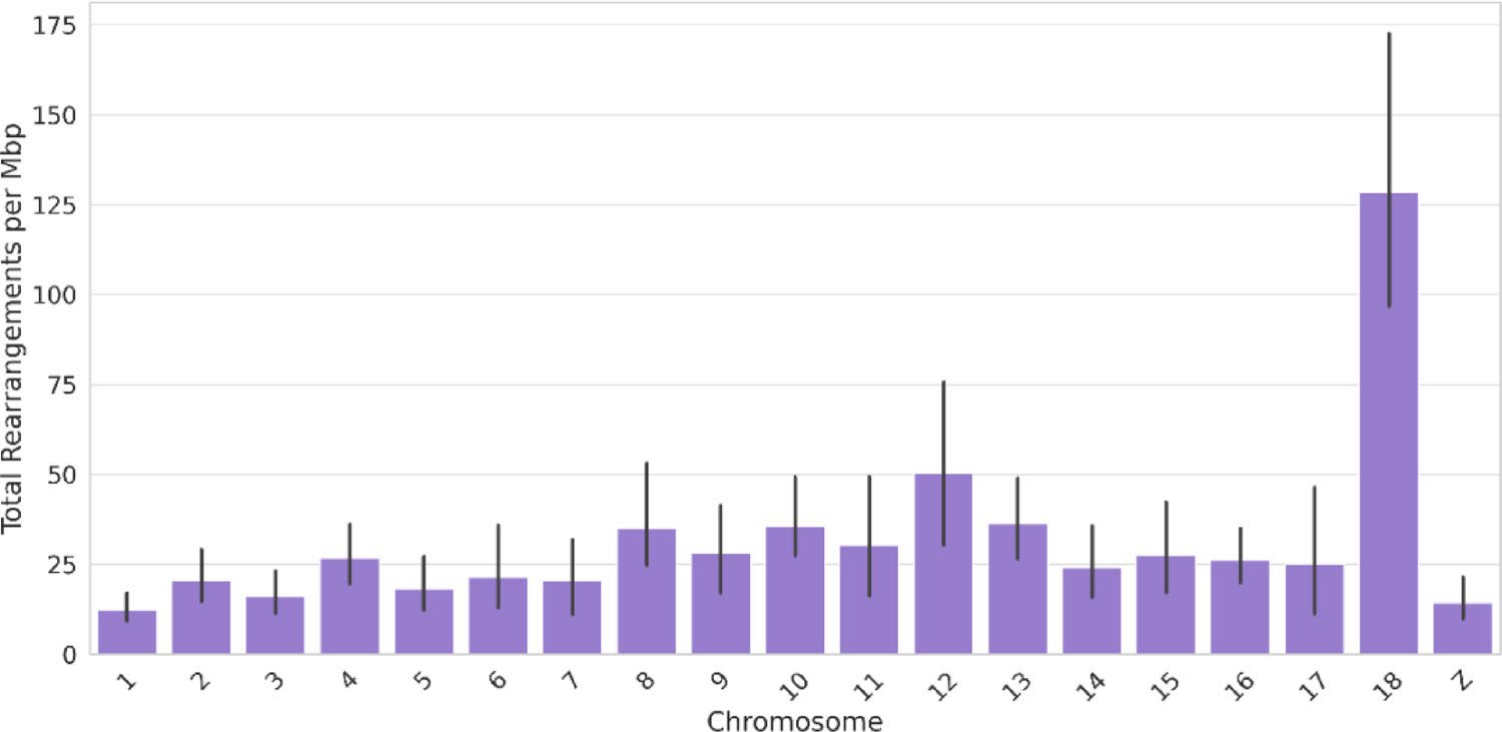
Mean value of structural variants (inversions, translocations, duplications, deletions) per Mbp for each chromosome from the syntenic analysis between *Podarcis* species and P. cretensis

The similarity scores of the genome alignments resulting from D-GENIES (Fig. 1) agree with the current phylogeny of wall lizards (Yang, Feiner et al. 2021), with the most closely related species having the highest similarity. Dot plots (Fig. 2) revealed that the most syntenic genomes were those between *P. cretensis* and *P. raffonei,* with the fewest inversions and translocations compared with the other genome comparisons. In contrast, the *genomes of P. melisellensis and P. muralis are less syntenic with the P. cretensis genome*, as there are extensive scattered matches and translocations. Most inventions are observed between *P. cretensis*—*P. filfolensis* and *P. cretensis*—*P. erhardii* comparisons. Chromosomes 8 and 12 presented the greatest structural variation in total number, whereas chromosome 17 presented the lowest, as it presented the fewest inversions, translocations, duplications, and deletions among all chromosomes. When normalized per Mbp (Fig. 3), chromosome 18 is the most structurally dynamic across all categories (inversions, translocations, duplications, and deletions).

### Comparative gene content and annotation quality across Podarcis genomes

Gene prediction revealed 22,861 protein-coding genes in *P. cretensis*, while those in published annotations of *P. muralis*, *P. raffonei*, and *P. lilfordi* were 21,258, 20,895, and 25,663, respectively. BUSCO completeness of the gene annotation was 94.8% for *P. cretensis,* with other gene annotations ranging from 94.7% (*P. lilfordi*) to 98.4% (*P. raffonei*). Compared with the other species, *P. cretensis* presented the highest percentage of single-copy genes and the lowest percentage of duplicated genes. RepeatMasker showed that approximately 45.9% of the genome assembly is composed of repetitive elements, most of them retrotransposons, including 15.2% long interspersed nuclear elements (LINEs), the highest among *Podarcis* (Table 2). Gene density was calculated 14.6, similar to *P. muralis* (Table 3), while cds/gene ratio is 19.46. Transcripts per gene are similar between *Podarcis* except *P. raffonei* where the ratio is much higher (Table 2).

**Table 2:** Repeat content comparison between *Podarcis* species

Repeat Class	P. cretensis	P. raffonei (rPodRaf1)	P. muralis (PodMur1.0)	P. lilfordi (rPodLil1.2)
Total repeats	45.9%	48.2%	37.6%	38.7%
SINEs	2.7%	0.1%	4.7%	4.0%
LINEs	15.2%	9.2%	12.1%	12.8%
LTR elements	3.77%	16.75%	1.4%	2.4%
DNA transposons	3.38%	15.07%	7.3%	7.2%
Unclassified	19.6%	5.5%	12.0%	12.3%
Total interspersed repeats	44.65%	48.2%	37.6%	38.7%

**Table 3:** *Podarcis* annotations comparison

Species	Gene density	cds/gene	GC content (%)	Transcript/gene
	14.60	19.46	44.10	1.64
*P. lilfordi*	17.58	17.59	43.72	1.70
*P. muralis*	14.60	25.46	45.33	1.69
*P. raffonei*	17.67	32.09	43.90	3.45

### Microchromosomes and sex chromosomes

**Fig. 4:**
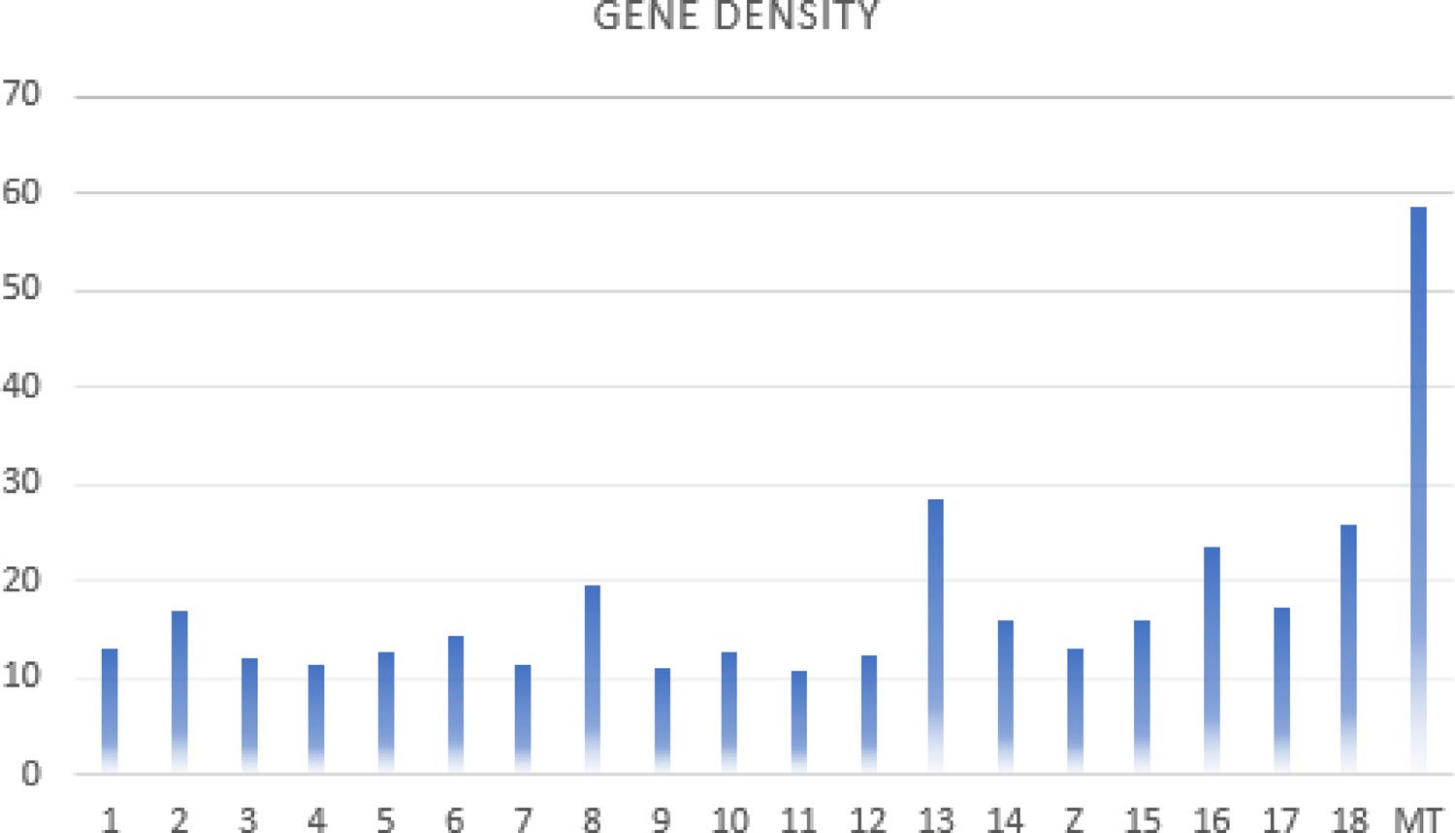
Gene density per chromosome of P. cretensis

Based on synteny analysis, GC content, and gene density metrics the existence of one microchromosome pair (Chr18) was confirmed, in accordance to most species of Lacertini (Naveira, Rojo et al. 2023). Chr18 is the smallest chromosome, with a size of ~ 14,6 Mbp in *P. cretensis*, while the remaining chromosomes range gradually from ~ 141,6 Mbp to ~ 43,6 Mbp. It has the highest GC content (50%) and ranks second in gene density among the *P. cretensis* chromosomes (Fig. 4), with 375 genes in total. The same pattern of gene density and GC content was also observed in the other *Podarcis*. Synteny analysis revealed that Chr18 has the largest number of rearrangements per Mbp (Fig. 3). Among the genes of the Chr18 of *P. cretensis*, are stress response genes such as HSPs and CIRBP, housekeeping genes such as eEF2 and NDUFA13, and fertilization-related genes such as IZUMO4 and Zona pellucida sperm-binding gene 4 (ZP4) are present.

The Z chromosome, which is a sex chromosome, was assembled into one scaffold of 51,4 Mbp in length, similar to that of other *Podarcis* (Gomez-Garrido, Cruz et al. 2023) and almost the same length as the Z chromosomes of *P. gaigeae* (https://www.ncbi.nlm.nih.gov/datasets/genome/GCA_964106915.2/) and *P. raffonei* (Gabrielli, Benazzo et al. 2023). The greatest structural differences in the synteny analysis of the Z chromosomes were observed between *P. cretensis* and *P. muralis*. It contains 676 genes crucial for gametogenesis, sexual differentiation, and reproductive success, such as vomeronasal type-2 receptor 26-like, neuronal PAS domain protein 2, androgen receptor (AR), spermatid perinuclear RNA-binding protein genes and ZP4.

### Gene conservation and species-specific features across Podarcis genomes

**Fig. 5:**
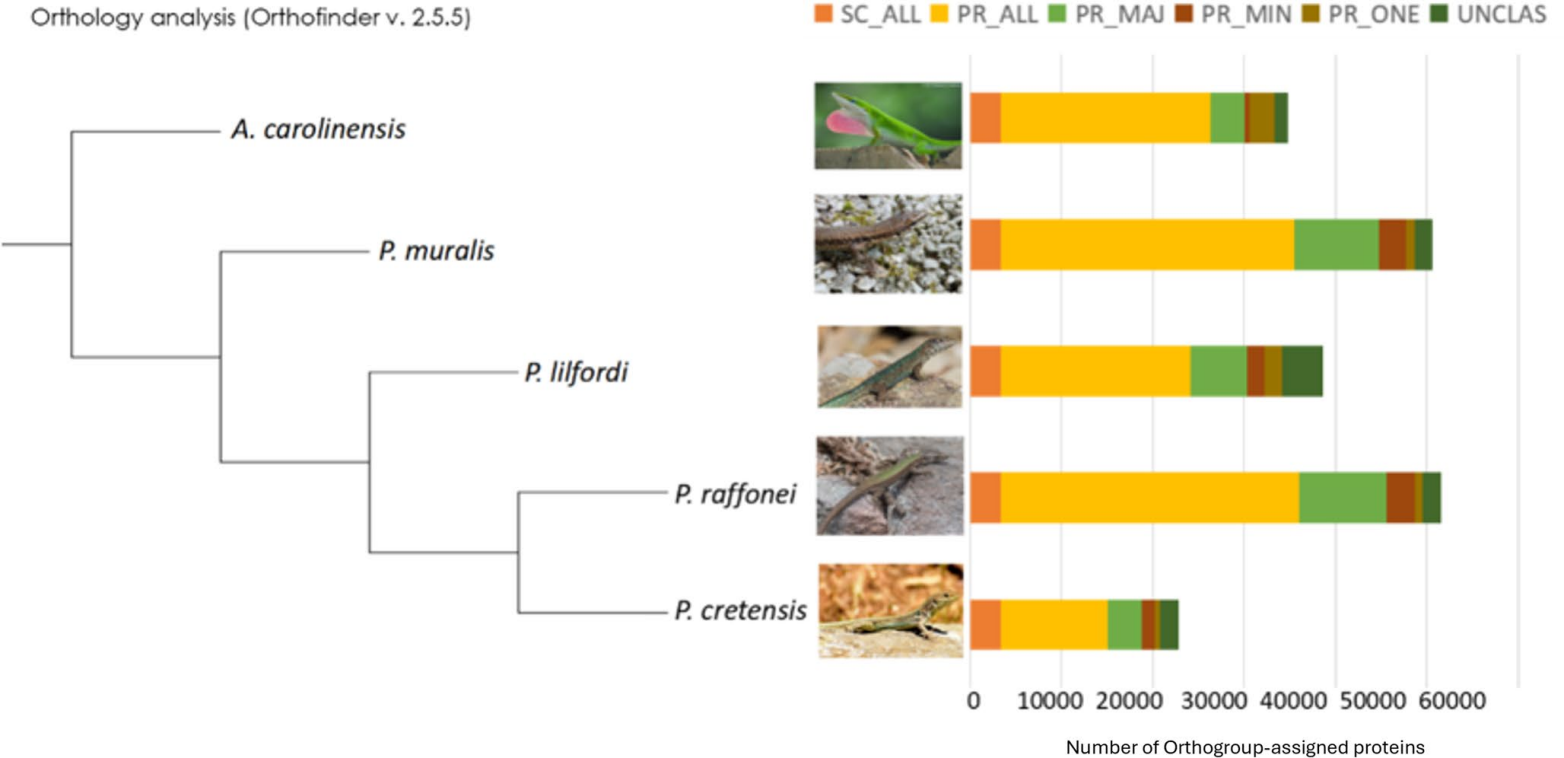
Distribution of the different orthogroups between Podarcis spp. Abbreviations used: SC_ALL, single-copy in all species; PR_ALL, present in all species (regardless of copy number); PR_MAJ, present in three or more species (majority); PR_MIN, present in one or two species (minority); PR_ONE, present in only one species; UNCLAS, not classified in any orthogroup. Photos of the species were obtained from https://www.inaturalist.org/

OrthoFinder assigned 186,530 predicted proteins (94.0% of the total) to 24,052 orthogroups. Fifty percent of all proteins were in orthogroups with nine or more proteins and were contained in the largest 6,482 orthogroups. There were 14,189 orthogroups that contained at least one protein from each species, 3,342 of which were single-copy proteins in all species (exactly one protein in each species). The proteins were classified into six categories on the basis of the conservation of the orthogroup (Fig. 5). Most of the proteins were found at varying copy numbers in all species (PR_ALL), whereas many species-specific proteins (PR_ONE) were unassigned or duplicated in only one species. In *P. cretensis*, there are 2,669 species-specific and unassigned proteins. BLAST analysis identified 709 proteins in the latter categories, 39% of which were classified as uncharacterized proteins. Transposons accounted for 10% of the elements as a result of not being removed from the repeat identification pipeline because they were not repetitive enough, whereas virus-related proteins accounted for 0.56%. Some notable genes in unassigned/species-specific categories are vomeronasal type-2 receptor 26-like genes, G-protein coupled receptor family genes, and interleukin and microcephalin genes.

### Gene expression overview

**Fig. 6:**
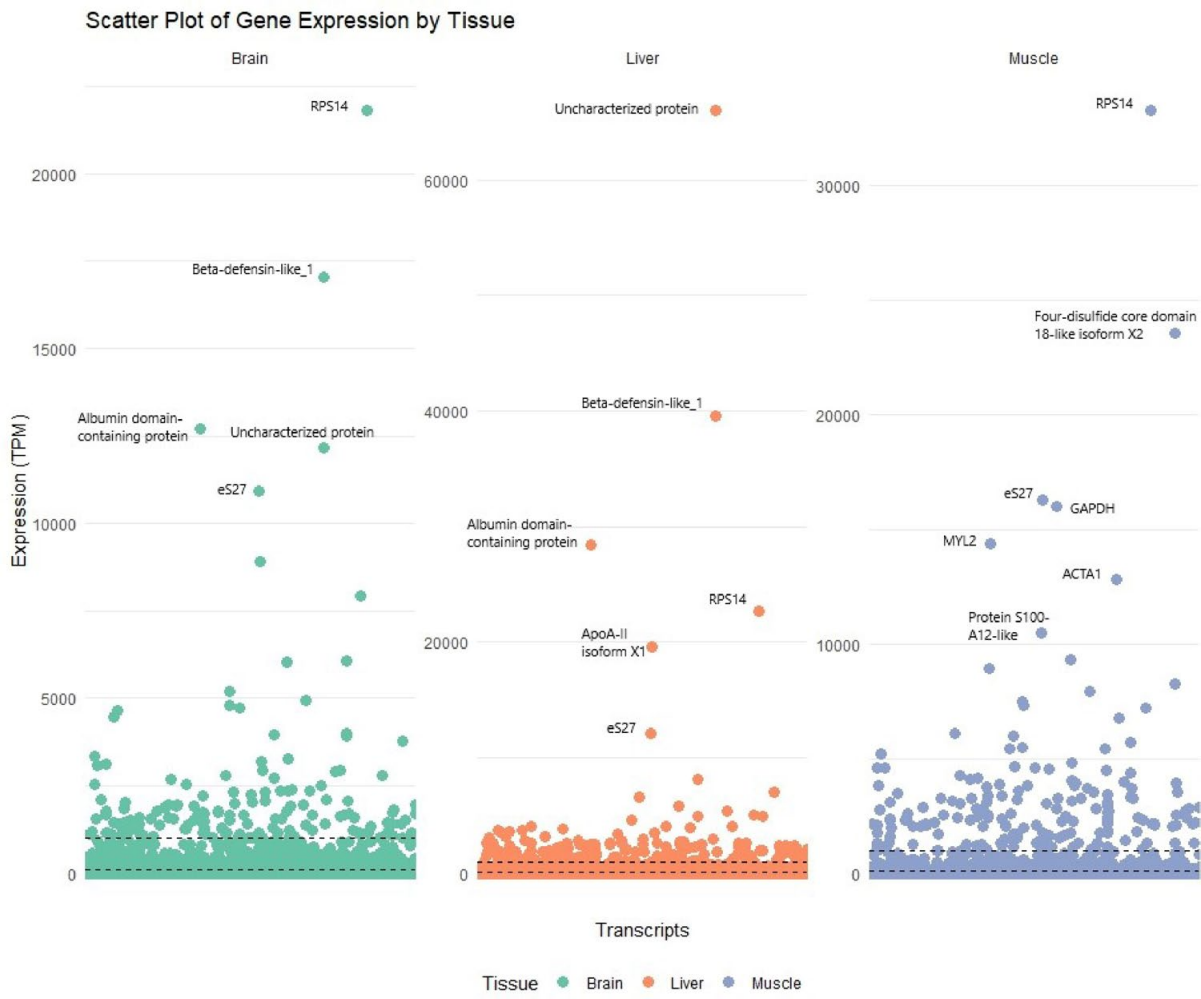
Scatterplot of gene expression in Transcripts per million (TPM) by tissue. The two dotted horizontal black lines represent the 100 and 1000 TPM thresholds. For transcripts with more than 10,000 TPMs is labelled the name of the corresponding gene

A total of 37,575 transcripts were quantified, and the expression levels of the predicted transcripts were plotted (Fig. 6). The expression analysis revealed that 89 genes were highly expressed (> 1,000 TPM) in all three tissues. Among them are genes related to ribosomal subunits (RPS14, RPS27, RPS28, RPL29, RPL42), Thymosin beta and cytochrome b-c1 complex subunit 10. The liver has relatively high expression levels of certain genes that express proteins involved in metabolic regulation, the immune response, and protein binding, such as Serpin domain-containing proteins, PSPH, and FABPs. In the brain, genes whose expression is upregulated compared with that in other tissues include FKBP12 (peptidyl-prolyl cis–trans isomerase FKBP1A), Complexin-1, mesotocin-neurophysin MT, Gamma-soluble NSF attachment protein, SNAP-25, CaMKII inhibitor 1, clathrin light chain, synaptotagmin-1, and hippocalcin. The genes upregulated in muscles compared with the brain and liver are the troponic C gene, fast skeletal myosin light chain 2 (MLC2) gene, phosphoglycerate mutase (PGAM) gene, troponin I gene, troponin T gene, galectin gene, keratin, type I cytoskeletal 13 (KRT13) gene and glycogen phosphorylase gene, among others. The transcripts with the highest expression in each tissue are shown in Fig. 6 and in more detail Supplementary Table SA4. Transcripts of genes of interest related to adaptation mechanisms and their expression levels are presented to Supplementary Table SA5.

## Discussion

This work focused on the analysis of the genome of the Cretan wall lizard *P. cretensis* and its genomic comparison with those of other lizards from the *Podarcis* genus. In addition, a large set of RNAseq data were generated and deposited in public repositories in an attempt to provide an initial characterization at *P. cretensis* gene expression levels across different conditions. The main findings revealed that *P. cretensis* and *P. raffonei* present the best genome assemblies and the most syntenic among the Podarcis species identified in this study while synteny analysis showed that Chr18 has the largest number of rearrangements. Moreover, *P. cretensis* presented the highest percentage of single-copy genes and the lowest percentage of duplicated genes. These duplicated genes are primarily associated with immune and sensory-related gene families, including chemokines, interleukins, immunoglobulin-like domain proteins, secreted proteins and vomeronasal type-2 receptors (V2R2).

### Genomes and gene set comparison

The genome sizes of species within the *Podarcis* genus were found to be relatively stable, ranging from 1,460 Mbp (*P. lilfordi*) to 1,510 Mbp (*P. muralis and P. raffonei*), with most genome assemblies examined presenting high completeness in terms of gene content as most of the genomes have > 95% complete BUSCO scores. The *P. cretensis* assembly is one of the highest quality and contiguous, with the lowest number of scaffolds and duplicated genes. In addition, the percentage of complete genes (97.8%) indicates a thorough and accurate annotation of the genome. *Podarcis cretensis* also has the highest k-mer completeness and consensus quality value (QV) among the corresponding values of the genomes of *P. lilfordi* and *P. raffonei*, thus further confirming the high quality of the current assembly and providing a consistent basis for reference in future studies.

Overall, the BUSCO scores for both the genome assemblies and the gene annotations, along with the contig N50, scaffold N50 and scaffold numbers, indicate that *P. cretensis* and *P. raffonei* present the best genome assemblies among the *Podarcis* species identified in this study. The high BUSCO completeness indicates that nearly all conserved single-copy orthologues are present, making both the created gene set and the genome valuable tools for comparative genomic studies.

For the gene set comparison, *P. raffonei* had the highest BUSCO completeness (98.4%), while the percentages of duplicated BUSCOs in the *P. muralis* and *P. raffonei* gene sets were greater than those in the other species, a result that was also confirmed via orthology analysis. The CDS per gene ratio which often used as quality meter of an annotation, in eukaryotes is expected to be greater than 1 as genes contain introns. However, a high ratio may indicate fragmentation rather than high quality assembly. *Podarcis raffonei* high CDS/gene and transcripts/gene ratio can be partially explained from the high gene duplication number (Table 1) which can affect gene structure and transcript diversity. In general, the high number of duplications could reflect some expansion in certain gene families, which could be advantageous under specific ecological pressures or adaptive processes. This can possibly an explanation in the case of *P. raffonei*, as it is a narrow endemic species found only on four small islands (Aeolian Islands). Nevertheless, the protein coding number fluctuates at similar levels between the available *Podarcis* annotations. In *P. cretensis* the most duplicated orthogroups included genes involved in gene expression, such as zinc finger protein genes and Musashi RNA binding protein genes; in neuronal function and synaptic regulation, such as RIMS1; and in microtubule-associated protein genes; and in cellular signalling and stress responses, such as serine/threonine kinase (Tssk) genes and protein-tyrosine phosphatase (PTP) genes. Tssk and PTP proteins have been studied across various organisms, where they are involved in spermatogenesis (Xue, Zhang et al. 2021), differentiation (Yang, Wang et al. 2022), the immune response (Mustelin, Vang et al. 2005, Perez-Quintero, Abidin et al. 2024), the stress response and adaptation to environmental changes (van der Velden, Wang et al. 2011). Moreover, we identified interesting duplications in *P. cretensis*, particularly in gene families related to immune and sensory functions such as chemokines, interleukins, Ig-like domein proteins, secreted proteins and vomeronasal type 2 receptors (V2R2). The latter are found in a variety of jawed vertebrates (Zhang, Sakuma et al. 2022) and are related to the detection of water-soluble peptides and the control of pheromone-induced male‒male aggression (Brykczynska, Tzika et al. 2013, Silva and Antunes 2017). The existence of multiple copies of these genes in *P. cretensis* could indicate a specific adaptation to unique ecological niches or social behaviours that occur on the island of Crete, but further work is needed to understand the exact evolutionary traits of this gene family.

### Synteny analysis between Podarcis

Even though *P. cretensis* is the closest relative to *P. erhardii* among the examined species (Yang, Feiner et al. 2021), synteny analysis revealed that its genome is more syntenic with that of *P. raffonei* (Fig. 2). The high synteny between *P. cretensis* and *P. raffonei* can be partially explained by the fact that both species are Mediterranean island endemics; as a result, they experienced reduced gene flow with other populations and experienced less recombination-driven genomic shuffling due to their smaller population sizes, whereas *P. erhardii* is more widely distributed in the Aegean region (Psonis, Antoniou et al. 2021). Another reason for this could also be the genome assembly and annotation of the species, which is a common problem in synteny analyses, as poor genome assembly quality can be a source of error when detecting synteny (Liu, Hunt et al. 2018, Steenwyk and King 2024). Newly assembled genomes tend to have better quality than those from the previous decade. Among *Podarcis* the most high-quality assemblies were those of *P. cretensis* and *P. raffonei*, which could result in better synteny results. D-GENIES results showed that the most structural variants across species were detected in Chr18 of *Podarcis* (Figure), with Chr12 and Chr8 showed the highest number of duplications and translocations. This implies that they might have experienced distinct evolutionary modifications, perhaps as a result of gene expansion, recombination, or changes in gene regulation. Chr17, on the other hand, has the lowest number of rearrangements overall, suggesting that it is a highly functionally stable and conserved region that has probably been preserved by natural selection.

### Microchromosomes

The presence or absence of microchromosomes has had a profound impact on genome evolution and architecture across vertebrates (Srikulnath 2013, Perry, Schield et al. 2021, Srikulnath, Ahmad et al. 2021). In lineages that retain microchromosomes, such as most lizards and birds, the genome remains partitioned into many small, gene-rich units, whereas in lineages that lost them (like mammals, crocodiles, and geckos), those gene packages have been redistributed onto fewer, larger chromosomes (Waters, Patel et al. 2021). Even if there is not a universal definition of what a microchromosome is, it presents distinct characteristics compared with macrochromosomes, including higher GC content, increased gene density, few transposable elements, a unique nuclear architecture (Perry, Schield et al. 2021, Srikulnath, Ahmad et al. 2021) and, several times, a size discontinuity from the previous larger chromosome rather than a gradual decrease in size. Usually, a < 30 Mb cut-off is used for the original karyotypic “definition” of a microchromosome (Pinto et al. 2023). Chromosome 18 of the examined across all examined *Podarcis* genomes fulfils most of these criteria. Synteny analysis revealed that there are more structural rearrangements between chromosome 18 of *P. cretensis* and the other chromosomes of the same species, suggesting possible important biological interpretations. The same pattern has also been observed in blacktail brush lizards (*Urosaurus nigricauda*), where some microchromosomes with high repeat content are more prone to rearrangements than macrochromosomes are (Davalos-Dehullu, Baty et al. 2023). Other studies have shown that these gene-enriched areas usually include housekeeping genes and other broadly expressed genes. In birds, for example, microchromosomes have been shown to contain many housekeeping genes and to have strong interchromosomal interactions related to active gene transcription (Liu, Wang et al. 2021a), but in lizards, there is a need for more evidence concerning microchromosome gene content (Deakin and Ezaz 2019). The presence of genes involved in the immune system, stress response, fertilization, metabolism and neurotransmission in the microchromosomes of *P. cretensis* could suggest that these microchromosomes not only are evolutionary remnants but also actively shape important biological traits.

### Gene families of interest related to adaptation mechanisms

In vertebrate ectotherms, stress-related responses at the genome level are associated with heat shock protein genes (HSPs), cold shock protein genes, hypoxia-related genes such as HIFs, genes involved in apoptosis, “zombie” genes (Wollenberg Valero, Garcia‐Porta et al. 2022) and genes related to cancer (Pozhitkov, Neme et al. 2017, Hayes, Dinkova-Kostova et al. 2020). In the remaining section, we will mention relative changes in gene expression. However, since we have no replicates for each condition, we cannot statistically verify these changes. Nevertheless, we mention these changes because they might hint on true expression differences and could thus help to guide functional experiments on these genes.

HSPs usually act as molecular chaperones, assisting organisms in managing stress (Sørensen, Kristensen et al. 2003). In particular, Hsp70 is involved in transporting proteins across cellular membranes and safeguarding neurons from apoptosis (Gething and Sambrook 1992, Mailhos, Howard et al. 1994, Feder and Hofmann 1999). The Hsp90 family serves as chaperones for steroid and hormone receptors, plays a role in cell signalling, and contributes to the myelination that protects neuronal cells (Pratt 1998, Tedeschi, Kennington et al. 2015).

In *P. cretensis*, we detected increased expression of both Hsp70 and Hsp90 in brain tissue of individuals in Mikronisi and Theriso, but not in the brains of individuals from the Lefka Ori Mountains (Supplementary Table SA5). This population is located at an altitude of 2000 m a.s.l. experiencing low night temperatures, as well as snow cover for more than 4 months per year. Interestingly, when individuals from this location acclimate to low-altitude conditions, the expression of these proteins is similar to that of other individuals from low-altitude populations. If this difference proves to be significant then it would suggest that the expression of HSPs is related to thermal adaptation over short time periods. Other studies have shown that for Hsp70, the active temperature range was more crucial for thermal adaptation to increased ambient temperatures in lizards than was their geographic location (Dang, Xu et al. 2018).

Transcriptomic results also revealed high levels of expression of the cold-inducible RNA-binding protein-encoding gene (CIRBP), especially in the brain. This cold shock protein is usually expressed in response to cold stress but it is much more studied in mammals, where it has been implicated in a variety of mechanisms, such as cold stress adaptation, cancer (Lujan, Ochoa et al. 2018), hypoxia and apoptosis (Liu, Liu et al. 2021b). Even in other reptiles, research on CIRBP is relatively limited, and studies have shown that CIRBP is expressed differentially in response to heat stress, especially in the brain (Akashi, Cádiz Díaz et al. 2016, He, Zhan et al. 2024). Interestingly, the response of amphibians to light signals has also been reported (Sugimoto and Jiang 2008). Orthology analysis revealed that CIRBP has orthologues in all the *Podarcis* species of our study. Importantly, there is only one copy of this gene in *P. cretensis,* but as many as three in *P. lilfordi* and four in P. *raffonei* four, indicating possible species-specific adaptations within the *Podarcis* group. Our findings also revealed high expression levels of this transcript in all three populations, suggesting that CIRBP may play a vital role in the stress response of *P. cretensis* and may help this species adapt to different thermal conditions.

Other important gene families, such as hypoxia-inducible factors (HIFs) and the peroxiredoxin family, are involved in adaptation to hypoxic conditions. HIF genes express transcription factors that play critical roles in the cellular response to low oxygen (hypoxia). They regulate the expression of numerous genes involved in processes such as angiogenesis, erythropoiesis, glucose metabolism, and cell survival (Majmundar, Wong et al. 2010; Semenza 2012). In reptiles, hypoxia stress can be induced by various factors, such as environmental conditions, high altitudes (Yang, Qi et al. 2014), inactivity (brumation) due to low ambient temperatures (Bickler and Buck 2007) and regeneration processes (Novianti, Juniantito et al. 2019). In *P. cretensis*, we detected relatively low expression of HIF-1, HIF-3 and hypoxia-inducible factor-proline dioxygenase (PHD), key enzymes involved in the regulation of the hypoxia-inducible factor (HIF) pathway, under different conditions. This could indicate that the hypoxia stress experienced by *P. cretensis* is limited under those conditions and, second, is not strongly related to altitude, which is expected, as the species is found at elevations no higher than 2100–2200 m. Moreover, EGLNs (EGLN1, EGLN3), which regulate HIFs (Strocchi, Reggiani et al. 2022), were expressed at very low levels in our screening, suggesting that the expression of HIFs may be an indirectly adaptation to handling stress before capture. Orthology analysis revealed that HIF-1α belongs to an orthogroup with only single-copy genes from each *Podarcis* species, indicating that this gene is highly conserved. Further analyses are needed to test and confirm these first observations.

Finally, we looked for transcripts related to enzymes and proteins for protection and restoration of the cells that have been detected in other reptiles. This category includes peroxiredoxins (PRDXs) and ERCC family genes. Our findings revealed that PRDX1, PRDX3 and PRDX4 are among the highly expressed genes under different conditions, suggesting that *P. cretensis* probably relies on its antioxidant defence mechanisms. On the other hand, repair genes such as ERCC6 presented relatively low expression levels. This gene is a type of DNA repair gene from the ERCC family and has been detected in other reptiles, such as Anan’s rock agama and *Thermophis baileyi* (Yan, Zhang et al. 2022), and may be associated with adaptation to UV radiation. PRDXs are multifunctional enzymes whose role is to protect cells from stress and preserve the homeostasis of many cellular processes. In addition, they serve as regulators of redox signalling, molecular chaperones, and proinflammatory factors, playing significant roles in oxidative defence, redox signalling pathways, protein folding, cell cycle regulation, DNA integrity, inflammation, and carcinogenesis (Wu, Deng et al. 2022). Organisms that frequently experience anoxic or ischemic conditions in their natural environment are often equipped with elevated baseline activities of these types of antioxidant enzymes (Storey 2006). In lizards, peroxiredoxins play major roles in both antioxidant defences (Manevich and Fisher 2005, Issartel, Hervant et al. 2009) and freeze tolerance (Voituron, Servais et al. 2006). The expression levels of the transcripts of the mentioned proteins are given in Supplementary Table SA5.

## Conclusion

The newly published high-quality genome of *P. cretensis* is a powerful tool for comparative genomic and transcriptomic studies, deepening our understanding of chromosome gene content, gene expression and genome evolution. The current study is the most extensive comparative genome analysis among *Podarcis* species, both at the genomic and transcriptomic levels, with an emphasis on *P. cretensis*. The increase in the number of high-quality and contiguous genome assemblies of reptiles will contribute significantly to a wide range of evolutionary aspects in this diverse group, providing more answers to key topics such as microchromosome evolution and function, as well as genotype—expression associations. The newly predicted gene set led to a first sight of the expression levels of various genes related to adaptation in different environments and tissue-specific expression profiling. Simultaneously, the comparative genomic approaches revealed gene associations between *Podarcis* species revealed gene families which are possibly involved in environmental adaptation, especially in insular endemic species such as *P. cretensis*, bringing the scientific community a step closer to understanding the different aspects of genomic adaptation of reptiles to environmental challenges.

## Supplementary Information

Below is the link to the electronic supplementary material.

Supplementary Material 1

## Data Availability

The sequencing reads are available from the Sequence Read Archive (SRA) under the BioProject accession number PRJNA1229138. The genome is available under the BioProject accession number PRJEB61853.
