# Supplementary material for: Unravelling the thread of Podarcis omics; insights into the genome and transcriptome of the Cretan wall lizard
Source: Genes Genomics. 2025 Oct 25;48(1):27–43. doi: 10.1007/s13258-025-01676-1 (PMC12860763; doi:10.1007/s13258-025-01676-1)
Supplement: Supplementary file 1 — Supplementary Material 1 [file 13258_2025_1676_MOESM1_ESM.docx]

Unravelling the thread of *Podarcis* omics; insights into the genome and transcriptome of the Cretan Wall Lizard.

**Journal: Genes & Genomics**

**Authors**

Manos Stratakis^1,2*^, Panagiotis Ioannidis^3^, Iliana Bista^4, 5^, Dominic Absolon^5^, Will Eagles^5^, Shane McCarthy^5^, Amy Denton^5^, Petros Lymberakis^2^, Nikos Poulakakis^1,2,3*^

^1^ Department of Biology, School of Sciences and Engineering, University of Crete, Heraklion, Greece

^2^ Natural History Museum of Crete, School of Sciences and Engineering, University of Crete, Heraklion, Greece

^3^ Institute of Computer Science (ICS), Foundation for Research and Technology – Hellas (FORTH), Heraklion, Greece

^4^Senckenberg Research Institute and Natural History Museum, 60325 Frankfurt am Main, Germany

^5^Wellcome Sanger Institute, Tree of Life, Wellcome Genome Campus, CB10 1SA, UK

*Corresponding authors
Manos Stratakis, email: [mastratakis79@gmail.com](mailto:mastratakis79@gmail.com), phone number: +306947929799
Nikos Poulakakis, email: [poulakakis@uoc.gr](mailto:poulakakis@uoc.gr), phone number: +306976264818



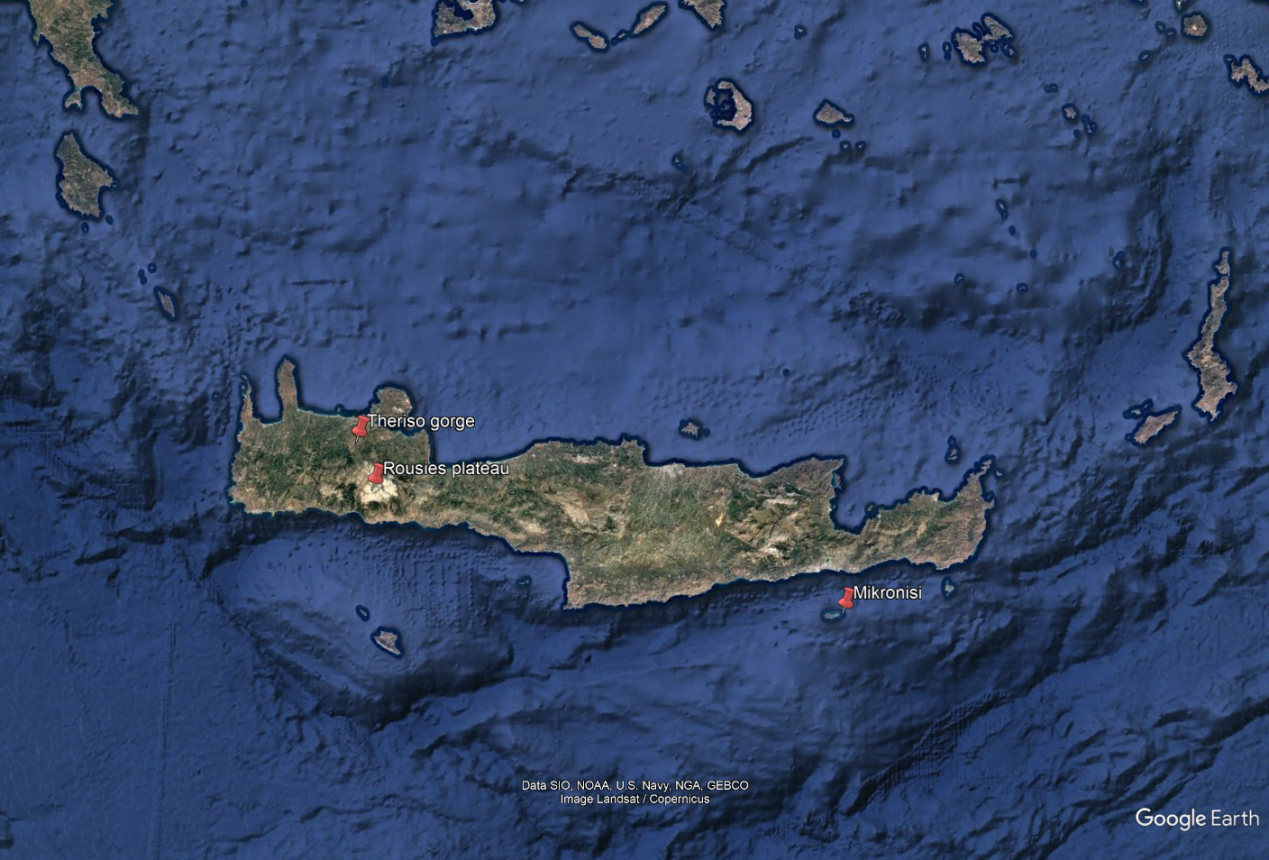


**Supplementary Figure SA1** Sampling sites for individuals used for RNA-seq data

**Supplementary Table SA1 RepeatMasker summary of P. cretensis assembly.**

|  | **Number of elements*** | **Length occupied (bp)** | **Percentage of sequence** |
| --- | --- | --- | --- |
| Retroelements | 1729167 | 326705752 | 21.67 |
| SINEs | 321292 | 40709876 | 2.70 |
| Penelope | 18834 | 3128786 | 0.21 |
| LINEs | 1275309 | 229149471 | 15.20 |
| CRE/SLACS | 0 | 0 | 0.00 |
| L2/CR1/Rex | 758168 | 130663446 | 8.67 |
| R1/LOA/Jockey | 307 | 49578 | 0.00 |
| R2/R4/NeSL | 0 | 0 | 0.00 |
| RTE/Bov-B | 329262 | 60211151 | 3.99 |
| L1/CIN4 | 160519 | 34542878 | 2.29 |
| LTR elements | 132566 | 56846405 | 3.77 |
| BEL/Pao | 0 | 0 | 0.00 |
| Ty1/Copia | 7474 | 6253191 | 0.41 |
| Gypsy/DIRS1 | 41816 | 27122584 | 1.80 |
| Retroviral | 11161 | 4243439 | 0.28 |
| DNA transposons | 384480 | 50904287 | 3.38 |
| Hobo-Activator | 103536 | 15373663 | 1.02 |
| Tc1-IS630-Pogo | 277200 | 34394801 | 2.28 |
| En-Spm | 0 | 0 | 0.00 |
| MULE-MuDR | 0 | 0 | 0.00 |
| PiggyBac | 474 | 101778 | 0.01 |
| Tourist/Harbinger | 445 | 28106 | 0.00 |
| Unclassified | 1670581 | 295586065 | 19.61 |
| Total interspersed repeats | N/A | 673196104 | 44.65 |
| Small RNA | 205268 | 24808063 | 1.65 |
| Satellites | 1906 | 225843 | 0.01 |
| Simple repeats | 404435 | 16363640 | 1.09 |
| Low complexity | 26620 | 1274216 | 0.08 |
| **Total masked bp** | **N/A** | **692195035** | **45.91** |

* Most repeats fragmented by insertions or deletions have been counted as one element.

**Supplementary Table SA2** RNAseq samples generated in the frame of this study. NHMC is the unique Natural History Museum Code for every sample in its deposits. RT represents room temperature.

| **SampleID** | **Tissue** | **Origin** | **Acclimate Habitat** | **Sex** | **‍Used for gene prediction?** | **NHMC** |
| --- | --- | --- | --- | --- | --- | --- |
| ‍2960_Brain | Brain | Rousies | RT | female | ‍Yes | NHMC80.3.51.2960 |
| ‍2960_Liver | Liver | Rousies | RT | female | ‍Yes | NHMC80.3.51.2960 |
| ‍2960_Muscle | Muscle | Rousies | RT | female | ‍Yes | NHMC80.3.51.2960 |
| ‍2980_Brain | Brain | Theriso | RT | male | ‍Yes | NHMC80.3.51.2980 |
| ‍2980_Liver | Liver | Theriso | RT | male | ‍Yes | NHMC80.3.51.2980 |
| ‍2980_Muscle | Muscle | Theriso | RT | male | ‍Yes | NHMC80.3.51.2980 |
| ‍2996_Brain | Brain | Rousies | RT | male | ‍Yes | NHMC80.3.51.2996 |
| ‍2996_Liver | Liver | Rousies | RT | male | ‍Yes | NHMC80.3.51.2996 |
| ‍2996_Muscle | Muscle | Rousies | RT | male | ‍Yes | NHMC80.3.51.2996 |
| ‍3015_Brain | Brain | Mikronisi | Mikronisi | female | ‍Yes | NHMC80.3.51.3015 |
| ‍3015_Liver | Liver | Mikronisi | Mikronisi | female | ‍Yes | NHMC80.3.51.3015 |
| ‍3015_Muscle | Muscle | Mikronisi | Mikronisi | female | ‍Yes | NHMC80.3.51.3015 |
| ‍3010_Brain | Brain | Theriso | RT | female | ‍Yes | NHMC80.3.51.3010 |
| ‍3010_Liver | Liver | Theriso | RT | female | ‍Yes | NHMC80.3.51.3010 |
| ‍3010_Muscle | Muscle | Theriso | RT | female | ‍Yes | NHMC80.3.51.3010 |
| ‍3020_Brain | Brain | Mikronisi | coldroom | male | ‍Yes | NHMC80.3.51.3020 |
| ‍3020_Liver | Liver | Mikronisi | coldroom | male | ‍Yes | NHMC80.3.51.3020 |
| ‍3020_Muscle | Muscle | Mikronisi | coldroom | male | ‍Yes | NHMC80.3.51.3020 |
| ‍3023_Brain | Brain | Mikronisi | coldroom | female | ‍Yes | NHMC80.3.51.3023 |
| ‍3023_Liver | Liver | Mikronisi | coldroom | female | ‍Yes | NHMC80.3.51.3023 |
| ‍3023_Muscle | Muscle | Mikronisi | coldroom | female | ‍Yes | NHMC80.3.51.3023 |
| ‍3024_Brain | Brain | Mikronisi | Mikronisi | male | ‍Yes | NHMC80.3.51.3024 |
| ‍3024_Liver | Liver | Mikronisi | Mikronisi | male | ‍Yes | NHMC80.3.51.3024 |
| ‍3024_Muscle | Muscle | Mikronisi | Mikronisi | male | ‍Yes | NHMC80.3.51.3024 |
| 2994_Brain | Brain | Rousies | Rousies | female | ‍Yes | NHMC80.3.51.2994 |
| 2994_Liver | Liver | Rousies | Rousies | female | ‍Yes | NHMC80.3.51.2994 |
| ‍2994_Muscle | Muscle | Rousies | Rousies | female | ‍Yes | NHMC80.3.51.2994 |
| ‍3003_Brain | Brain | Rousies | Rousies | male | ‍Yes | NHMC80.3.51.3003 |
| 3003_Liver | Liver | Rousies | Rousies | male | ‍Yes | NHMC80.3.51.3003 |
| 3003_Muscle | Muscle | Rousies | Rousies | male | ‍Yes | NHMC80.3.51.3003 |
| 2976_Brain | Brain | Theriso | Theriso | female | ‍Yes | NHMC80.3.51.2976 |
| 2976_Liver | Liver | Theriso | Theriso | female | ‍Yes | NHMC80.3.51.2976 |
| 2976_Muscle | Muscle | Theriso | Theriso | female | ‍Yes | NHMC80.3.51.2976 |
| 2975_Brain | Brain | Theriso | Theriso | male | ‍Yes | NHMC80.3.51.2975 |
| 2975_Liver | Liver | Theriso | Theriso | male | ‍Yes | NHMC80.3.51.2975 |
| ‍2975_Muscle | Muscle | Theriso | Theriso | male | ‍Yes | NHMC80.3.51.2975 |

**Supplementary Table SA3** Number of structural variants per species in comparison with the P. cretensis genome based on synteny analysis

| Species | Inversions | Translocations | Duplications | Deletions |
| --- | --- | --- | --- | --- |
| *P. erhardii* | 7909 | 11306 | 16001 | 7890 |
| *P. filfolensis* | 5707 | 5401 | 10771 | 5179 |
| *P. gaigeae* | 6132 | 6434 | 12246 | 5915 |
| *P. lilfordi* | 6483 | 6961 | 13711 | 6675 |
| *P. melisellensis* | 10106 | 15171 | 21083 | 10487 |
| *P. muralis* | 18288 | 20212 | 36269 | 17919 |
| *P. pityusensis* | 6690 | 8202 | 13508 | 6575 |
| *P. raffonei* | 5769 | 5040 | 10590 | 5010 |

**Supplementary Table SA4** The 20 transcripts with the highest expression levels in each tissue according to the UniRef50 database

| **seqid** | **Liver** | **seqid** | **Brain** | **seqid** | **Muscle** |
| --- | --- | --- | --- | --- | --- |
| g71.t1 | Small ribosomal subunit protein uS14 | g4434.t1 | Beta-defensin-like 1 | g71.t1 | Small ribosomal subunit protein uS14 |
| g17091.t1 | Albumin domain-containing protein | g17091.t1 | Albumin domain-containing protein | g21788.t1 | Glyceraldehyde-3-phosphate dehydrogenase |
| g4434.t1 | Beta-defensin-like 1 | g4433.t1 | Uncharacterized protein | g4944.t1 | Actin, alpha skeletal muscle |
| g20932.t1 | apolipoprotein A-II isoform X1 | g71.t1 | Small ribosomal subunit protein uS14 | g20852.t1 | Small ribosomal subunit protein eS27 |
| g4433.t1 | Uncharacterized protein | g20932.t1 | apolipoprotein A-II isoform X1 | g17457.t1 | Fructose-bisphosphate aldolase |
| g20852.t1 | Small ribosomal subunit protein eS27 | g6774.t1 | Thymosin beta | g17501.t1 | Fast skeletal myosin light chain 2 |
| g6774.t1 | Thymosin beta | g21897.t1 | Peptidase S1 domain-containing protein | g8514.t1 | Four-disulfide core domain 18-like isoform X2 |
| g22613.t1 | Small ribosomal subunit protein eS28 | g9232.t1 | Colipase | g22613.t1 | Small ribosomal subunit protein eS28 |
| g7962.t1 | Transferrin-like domain-containing protein | g22613.t1 | Small ribosomal subunit protein eS28 | g8506.t2 | Troponin C, skeletal muscle |
| g3265.t1 | 60S ribosomal protein L29 (Fragment) | g7962.t1 | Transferrin-like domain-containing protein | g19082.t2 | Globin domain-containing protein |
| g322.t1 | Serpin domain-containing protein | g12075.t1 | Metallothionein | g6774.t1 | Thymosin beta |
| g19082.t2 | Globin domain-containing protein | g19634.t1 | Large ribosomal subunit protein eL42 | g3265.t1 | 60S ribosomal protein L29 (Fragment) |
| g19634.t1 | Large ribosomal subunit protein eL42 | g19634.t1 | Large ribosomal subunit protein eL42 | g1956.t1 | Myosin light chain 1 skeletal muscle isoform |
| g5147.t1 | Small ribosomal subunit protein eS12 | g11803.t1 | interferon alpha-inducible protein 27-like protein 2A isoform X1 | g19634.t1 | Large ribosomal subunit protein eL42 |
| g20155.t1 | Phosphoserine phosphatase | g3265.t1 | 60S ribosomal protein L29 (Fragment) | g4400.t1 | Uncharacterized protein |
| g21897.t1 | Peptidase S1 domain-containing protein | g22780.t1 | cytochrome b-c1 complex subunit 10 | g5147.t1 | Small ribosomal subunit protein eS12 |
| g22780.t1 | cytochrome b-c1 complex subunit 10 | g19082.t2 | Globin domain-containing protein | g4398.t1 | Uncharacterized protein |
| g11803.t1 | interferon alpha-inducible protein 27-like protein 2A isoform X1 | g15.t2 | Protein AMBP | g5882.t1 | Globin domain-containing protein |
| g21101.t1 | FAU ubiquitin like and ribosomal protein S30 fusion | g12495.t1 | pancreatic elastase II | g19091.t1 | Globin domain-containing protein |
| g5882.t1 | Globin domain-containing protein | g13457.t1 | Fibrinogen beta chain | g4397.t2 | Unknown |

**Supplementary Table SA5** The expression levels (in TPM) of the genes/transcripts are referred to in the discussion of “Gene families of interest related to adaptation mechanisms”. NHMC provides the last 4 digits of the NHMC code (80.3.51.).

| NHMC | 3005 | | | 3024 | | | 2994 | | | 3003 | | | 2976 | | | 2975 | | |
| --- | --- | --- | --- | --- | --- | --- | --- | --- | --- | --- | --- | --- | --- | --- | --- | --- | --- | --- |
| Condition | Mikronisi | | | Mikronisi | | | Lefka Ori | | | Lefka Ori | | | Theriso | | | Theriso | | |
| Origin | Mikronisi | | | Mikronisi | | | Lefka Ori | | | Lefka Ori | | | Theriso | | | Theriso | | |
| Sex | Female | | | Male | | | Female | | | Male | | | Female | | | Male | | |
| Tissue | Brain | Liver | Muscle | Brain | Liver | Muscle | Brain | Liver | Muscle | Brain | Liver | Muscle | Brain | Liver | Muscle | Brain | Liver | Muscle |
| g202.t1/HSP70 | 45 | 1 | 33 | 87 | 5 | 59 | 3 | 135 | 32 | 3 | 70 | 78 | 40 | 2 | 81 | 42 | 291 | 169 |
| g266.t1/HSP90 | 827 | 85 | 79 | 824 | 156 | 37 | 32 | 576 | 62 | 13 | 515 | 45 | 1026 | 12 | 167 | 155 | 782 | 280 |
| g19467.t1/PGK1 | 474 | 203 | 325 | 588 | 373 | 320 | 195 | 554 | 801 | 143 | 617 | 251 | 671 | 267 | 1012 | 181 | 641 | 711 |
| g7251.t1/ERCC6 | 12 | 2 | 4 | 11 | 6 | 9 | 2 | 6 | 3 | 2 | 9 | 3 | 19 | 3 | 11 | 2 | 13 | 11 |
| g11272.t1/HIF3 | 14 | 0 | 3 | 8 | 10 | 0 | 2 | 1 | 8 | 6 | 9 | 4 | 6 | 1 | 6 | 1 | 12 | 7 |
| g11272.t2/HIF3 | 0 | 0 | 6 | 3 | 0 | 9 | 9 | 5 | 0 | 0 | 0 | 2 | 0 | 0 | 0 | 0 | 0 | 0 |
| g11272.t3/HIF3 | 0 | 0 | 0 | 0 | 0 | 0 | 0 | 0 | 0 | 0 | 0 | 0 | 0 | 0 | 0 | 0 | 0 | 0 |
| g180.t1/HIF1 | 42 | 1 | 14 | 47 | 19 | 27 | 12 | 27 | 19 | 16 | 37 | 18 | 43 | 12 | 25 | 11 | 34 | 28 |
| g180.t2HIF1 | 10 | 0 | 5 | 5 | 2 | 11 | 2 | 4 | 2 | 0 | 7 | 2 | 4 | 0 | 3 | 2 | 8 | 5 |
| g7272.t1/HIF1 | 39 | 0 | 43 | 26 | 11 | 42 | 1 | 24 | 15 | 6 | 34 | 9 | 14 | 4 | 12 | 6 | 48 | 26 |
| g7272.t2/HIF1 | 23 | 1 | 0 | 37 | 0 | 9 | 3 | 7 | 19 | 0 | 22 | 11 | 36 | 0 | 19 | 0 | 0 | 17 |
| g22709.t1/CIRBP | 784 | 21 | 81 | 573 | 45 | 67 | 67 | 1173 | 144 | 75 | 1494 | 75 | 2098 | 34 | 171 | 29 | 1779 | 89 |
| g518.t1/PHD | 0 | 7 | 3 | 1 | 9 | 3 | 2 | 1 | 4 | 2 | 1 | 3 | 0 | 0 | 7 | 0 | 0 | 11 |
| g9336.t1/PRDX1 | 81 | 280 | 59 | 121 | 272 | 41 | 174 | 177 | 68 | 201 | 162 | 36 | 141 | 342 | 97 | 209 | 107 | 214 |
| g19158.t1/PRDX1 | 271 | 53 | 67 | 309 | 24 | 154 | 28 | 425 | 203 | 36 | 468 | 131 | 444 | 34 | 281 | 76 | 433 | 208 |
| g7681.t1/PRDX3 | 108 | 139 | 57 | 86 | 80 | 83 | 134 | 113 | 132 | 64 | 89 | 78 | 116 | 150 | 85 | 92 | 136 | 64 |
| g6713.t1/PRDX4 | 264 | 791 | 263 | 156 | 420 | 133 | 335 | 153 | 124 | 358 | 185 | 145 | 125 | 428 | 192 | 355 | 146 | 111 |
| g6713.t2/PRDX4 | 2 | 3 | 18 | 29 | 0 | 6 | 2 | 8 | 0 | 0 | 0 | 1 | 0 | 0 | 0 | 4 | 8 | 14 |
|  |  |  |  |  |  |  |  |  |  |  |  |  |  |  |  |  |  |  |
| NHMC | 3020 | | | 3023 | | | 2960 | | | 2996 | | | 3010 | | | 2980 | | |
| Condition | Coldroom | | | Coldroom | | | RT | | | RT | | | RT | | | RT | | |
| Origin | Mikronisi | | | Mikronisi | | | Lefka Ori | | | Lefka Ori | | | Theriso | | | Theriso | | |
| Sex | Male | | | Female | | | Female | | | Male | | | Female | | | Male | | |
| Tissue | Brain | Liver | Muscle | Brain | Liver | Muscle | Brain | Liver | Muscle | Brain | Liver | Muscle | Brain | Liver | Muscle | Brain | Liver | Muscle |
| g202.t1/HSP70 | 474 | 10 | 75 | 353 | 7 | 55 | 225 | 9 | 19 | 101 | 5 | 18 | 110 | 11 | 28 | 75 | 2 | 15 |
| g266.t1/HSP90 | 566 | 144 | 164 | 353 | 95 | 76 | 616 | 186 | 142 | 573 | 89 | 107 | 451 | 133 | 157 | 411 | 86 | 87 |
| g19467.t1/PGK1 | 439 | 242 | 643 | 446 | 187 | 878 | 267 | 237 | 238 | 575 | 183 | 213 | 484 | 233 | 381 | 351 | 257 | 562 |
| g7251.t1/ERCC6 | 12 | 0 | 3 | 10 | 7 | 1 | 8 | 4 | 3 | 10 | 1 | 4 | 7 | 5 | 5 | 8 | 5 | 3 |
| g11272.t1/HIF3 | 5 | 0 | 0 | 2 | 11 | 0 | 5 | 5 | 0 | 3 | 0 | 2 | 2 | 6 | 7 | 9 | 0 | 3 |
| g11272.t2/HIF3 | 0 | 3 | 7 | 0 | 0 | 0 | 0 | 1 | 0 | 0 | 1 | 0 | 0 | 1 | 2 | 2 | 0 | 0 |
| g11272.t3/HIF3 | 0 | 0 | 0 | 0 | 0 | 5 | 0 | 0 | 0 | 0 | 3 | 0 | 0 | 0 | 3 | 0 | 0 | 0 |
| g180.t1/HIF1 | 44 | 42 | 15 | 35 | 25 | 9 | 30 | 8 | 11 | 25 | 13 | 9 | 26 | 11 | 9 | 20 | 14 | 22 |
| g180.t2/HIF1 | 19 | 16 | 13 | 22 | 19 | 7 | 7 | 3 | 4 | 10 | 2 | 5 | 4 | 3 | 15 | 10 | 6 | 7 |
| g7272.t1/HIF1 | 23 | 7 | 16 | 54 | 11 | 12 | 22 | 2 | 23 | 35 | 16 | 20 | 18 | 3 | 47 | 17 | 4 | 18 |
| g7272.t2/HIF1 | 62 | 20 | 37 | 6 | 39 | 31 | 24 | 11 | 18 | 0 | 0 | 8 | 29 | 12 | 26 | 30 | 15 | 45 |
| g22709.t1/CIRBP | 535 | 55 | 62 | 651 | 49 | 56 | 528 | 48 | 84 | 734 | 52 | 84 | 653 | 51 | 105 | 426 | 40 | 48 |
| g518.t1/PHD | 3 | 5 | 11 | 4 | 6 | 9 | 13 | 7 | 15 | 0 | 15 | 19 | 2 | 17 | 8 | 2 | 16 | 5 |
| g9336.t1/PRDX1 | 102 | 945 | 111 | 93 | 810 | 271 | 85 | 378 | 85 | 78 | 337 | 92 | 112 | 336 | 116 | 50 | 241 | 88 |
| g19158.t1/PRDX1 | 273 | 36 | 138 | 318 | 49 | 206 | 226 | 69 | 173 | 361 | 88 | 255 | 514 | 149 | 437 | 326 | 86 | 80 |
| g7681.t1/PRDX3 | 53 | 39 | 35 | 34 | 41 | 62 | 72 | 54 | 70 | 85 | 99 | 85 | 97 | 61 | 138 | 85 | 70 | 132 |
| g6713.t1/PRDX4 | 103 | 316 | 113 | 112 | 289 | 133 | 154 | 408 | 146 | 238 | 356 | 209 | 148 | 350 | 131 | 161 | 568 | 77 |
| g6713.t2/PRDX4 | 56 | 0 | 6 | 27 | 18 | 7 | 14 | 5 | 0 | 7 | 2 | 7 | 11 | 9 | 1 | 26 | 5 | 8 |

**Supplementary Table SA6** Orthofinder metrics

|  | All | *Anolis carolinensis* | *Podarcis cretensis* | *Podarcis lilfordi* | *Podarcis muralis* | *Podarcis raffonei* |
| --- | --- | --- | --- | --- | --- | --- |
| Number of genes | 198411 | 34813 | 22861 | 38622 | 50554 | 51561 |
| Number of genes in OGs | 186530 | 33286 | 20811 | 34191 | 48689 | 49553 |
| Number of unassigned genes | 11881 | 1527 | 2050 | 4431 | 1865 | 2008 |
| % of genes in OGs | 94.0 | 95.6 | 91.0 | 88.5 | 96.3 | 96.1 |
| % of unassigned genes | 6.0 | 4.4 | 9.0 | 11.5 | 3.7 | 3.9 |
| Number of OGs containing species | 24052 | 17138 | 17719 | 19584 | 21119 | 21349 |
| % of OGs containing species |  | 71.3 | 73.7 | 81.4 | 87.8 | 88.8 |
| Number of species-specific OGs | 1812 | 591 | 95 | 606 | 289 | 231 |
| Number of genes in species specific OGs | 7087 | 2670 | 619 | 1945 | 972 | 881 |
| % of genes in species specific OGs | 3.6 | 7.7 | 2.7 | 5.0 | 1.9 | 1.7 |
| G50 (assigned genes) | 9 |  |  |  |  |  |
| G50 (all genes) | 9 |  |  |  |  |  |
| O50 (assigned genes) | 5822 |  |  |  |  |  |
| O50 (all genes) | 6482 |  |  |  |  |  |
| Number of OGs with all species present | 14189 |  |  |  |  |  |
| Number of single copy OGs | 3342 |  |  |  |  |  |
